# Urine Dipstick Analysis on Automated Platforms: Is a Reliable Screening Tool for Proteinuria? An Experience from Umberto I Hospital in Rome

**DOI:** 10.3390/biomedicines11041174

**Published:** 2023-04-13

**Authors:** Sergio Terracina, Antonio Pallaria, Marco Lucarelli, Antonio Angeloni, Annarita De Angelis, Flavio Maria Ceci, Brunella Caronti, Silvia Francati, Giovanna Blaconà, Marco Fiore, Giampiero Ferraguti

**Affiliations:** 1 Department of Experimental Medicine, Sapienza University of Rome, 00185 Rome, Italy; sergio.terracina@uniroma1.it (S.T.); a.pallaria@uniroma1.it (A.P.); marco.lucarelli@uniroma1.it (M.L.); antonio.angeloni@uniroma1.it (A.A.); flaviomaria.ceci@uniroma1.it (F.M.C.); giovanna.blacona@uniroma1.it (G.B.); 2 Department of Human Neurosciences, Sapienza University Hospital of Rome, 00185 Rome, Italy; brunella.caronti@uniroma1.it; 3 Institute of Biochemistry and Cell Biology, IBBC—CNR, 00185 Rome, Italy

**Keywords:** albumin, kidney, urine analysis, creatinine, comparison between methods

## Abstract

**Simple Summary:**

The determination of albumin in the urine can give different results depending on the method used. Specialists must pay particular attention to the method used for the determination of urine albumin, creatinine, and ACR.

**Abstract:**

Urinalysis is commonly used as a screening tool for kidney disease. In many cases, the dipstick urine assay includes the assessment of albumin/protein and creatinine; consequently, the value of their ratio is available on the urine section report. Identification of albuminuria/proteinuria at early stages is an important issue to prevent or at least delay the onset of chronic kidney disease (CKD), kidney failure, and the progression of cardiovascular damage linked to the kidney’s loss of function. Sensitive and specific diagnostic methods are required for the assessment of such an important biomarker: urine albumin, creatinine, and their ratio (ACR) measured with quantitative assays are considered the gold standard. Routine dipstick methods (more rapid and at a lower cost) are intended for wide population screening. The aim of our study was to verify the reliability of an automated urinalysis dipstick method by comparing the results with the quantitative test of creatinine and albumin performed on a clinical chemistry platform. The first-morning voids of 249 patients who arrived from different departments were analyzed in the Central Laboratory of the University Hospital Policlinico Umberto I in Rome. We found a good correlation between the two assays, even though we observed that the dipstick assessment tends to overestimate the ACR’s value, disclosing a higher number of false positives if compared to the reference method. As an important novelty in this study, we analyzed our data considering age (starting from pediatric to geriatric patients) and sex as variables for a sub-stratification of the participants. Our results show that positive values need to be confirmed with quantitative methods, especially in women and younger people, and that from samples that resulted as diluted at the dipstick assay, the ACR’s values can be obtained if they are reanalyzed with quantitative assays. Moreover, patients with microalbuminuria (ACR 30–300 mg/g) or severe albumin urinary excretion (ACR > 300 mg/g) should be reanalyzed using quantitative methods to obtain a more reliable calculation of the ACR.

## 1. Introduction

Chronic kidney disease (CKD) or chronic kidney failure is a long-term condition characterized by gradual kidney function loss. Since diabetes and hypertension are the main risk factors for CKD, its prevalence is higher in countries with an elderly population [1,2,3]. Diabetes is involved in 30–50% of all CKD, concerning 285 million adults around the world. On the other hand, hypertension was assessed to affect more than 25% of the adult population. Moreover, the incidence of type 1 diabetes in children is rising in many countries; a 3.9% increase per year in the incidence of type 1 diabetes in children younger than 15 was revealed by the analysis of a European population-based registry in the years between 1989 and 2003, with the Scandinavian countries having the highest incidence, with 57 new cases reported per 100,000 children under 15 years of age per year [4].

A sensitive diagnostic method is important to identify patients with the early stages of kidney disease in order to prevent or at least delay its progression to kidney failure and the onset of cardiovascular damage related to the kidney’s loss of function [5,6]. An elevated loss of protein/albumin in urine is a marker of kidney damage; moreover, albuminuria has a direct toxic effect on the renal tubules [7].

The gold standard to assess protein loss through the kidneys is albuminuria/proteinuria, usually measured in a sample from the 24 h urine collection with a quantitative test on a chemical chemistry platform [8]. Results can be expressed as mg of albumin/protein in 24 h, per liter, or reported in grams of creatinine excreted. The assessment of urine albumin has several advantages compared to that of urinary total protein: a single protein can be detected by specific assays with more accuracy and precision; moreover, urine albumin is the most sensitive marker of protein loss because of compromised glomerular filtration [9].

Albumin-to-creatinine ratio (ACR) is important because it can fix the variance for a patient’s hydration if random or first-morning spot samples are used instead of the 24 h collection. Furthermore, this correlation is useful to correct the variance of albumin’s excretion due to the circadian rhythm [10]. The normal ACR range is estimated below 30 mg/g, while a moderate-severe excretion’s enhancement (microalbuminuria) is between 30 and 300 mg/g. An ACR above 300 mg/g is considered a severe increase in albumin excretion.

Increased albuminuria causes tubulointerstitial damage through the activation of proinflammatory mediators, leading to a progressive and permanent decline in renal function. Furthermore, albuminuria can predict the development of kidney damage and be used to stage chronic disease at its base. A urinary albumin excretion increase, and a pathological albuminuria range predict the development of cardiovascular and kidney damage and increase the risk of death in the diabetic and non-diabetic general population [11].

Albuminuria, creatinuria, and ACR can also be easily detected with specific semiquantitative dipstick assays. Compared to the quantitative methods of automated clinical chemistry analyzers, they result in faster and less expensive results, but with the major flaw of lower sensitivity [12]. In recent years, technological development has brought significant progress in automated urinalysis [13,14]. Complementary metal oxide semiconductor (CMOS) technology has enhanced analytical sensitivity and shows promise in microalbuminuria testing [15]. Meanwhile, quantitative reading of urinary test strips using reflectometry has improved, and microscopy-based urine particle analysis and its alternative, flow cytometry, have considerably progressed.

To improve results interpretation and enable the correction of urinary dilution, it has been studied the combination of dilution parameters (e.g., creatinine, specific gravity, and conductivity) in urine test strip readers and urine particle flow cytometers [13]. Automated urinalysis is useful for screening, diagnosing, and monitoring a broad variety of nephrological and urological conditions.

It has been suggested that a dipstick test result of “<1+” or less than trace has a high negative predictive value in the general community setting, with minimal risk of a missed diagnosis of macroalbuminuria [16]. At the same time, high false-positive rates emphasize the need for laboratory confirmation of positive results [17,18]. The aim of this retrospective study is to understand the reliability of the dipstick assay as a screening method for proteinuria.

## 2. Materials and Methods

**Figure 1:**
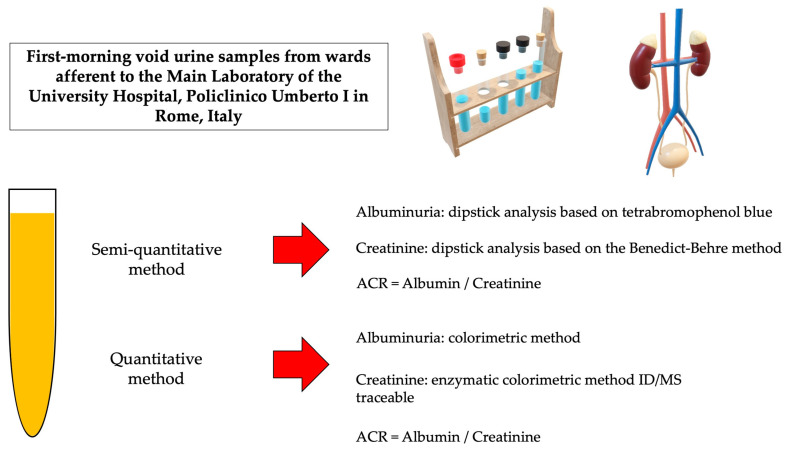
Sample preparation diagram. We aimed to compare two different diagnostic methods to evaluate kidney damage. From November 2020 to January 2021, we selected 249 sequential subjects of various ages and genders whose first morning void was analyzed at the Core Laboratory of the Policlinico Umberto I University Hospital in Rome. An initial semi-quantitative analysis was performed, followed by a quantitative test. Albumin was evaluated by dipstick analysis (Meditape UC-11A, Sysmex Corporation HQ: Kobe, Japan) based on a PH indicator (tetrabromophenol blue). The creatinine analysis was based on the Benedict-Behre method. The ACR, albumin creatinine ratio was calculated using the Sysmex UC-3500 Automatic Urine Analyzer’s semi-quantitative method. Quantitative analysis of albumin, creatinine, and total urinary protein were performed on a Cobas C 501 analyzer (Roche Diagnostics GmBH, Mannheim, Germany). Albumin was measured using a colorimetric method, creatinine by an enzymatic colorimetric method, and urinary proteins by a turbidimetric method. The ACR was calculated. Images have been created using the functionalities of Microsoft PowerPoint 365 Version 2112. https://www.microsoft.com/microsoft-365. Used with permission from Microsoft. Accessed on 13 March 2023.

In this retrospective study, we compared quantitative assays of urine albumin and creatinine on a chemical chemistry platform with a semiquantitative assessment performed on an automated system through a dipstick evaluation using the first-morning voids of 249 patients analyzed in the Central Laboratory of the University Hospital Policlinico Umberto I in Rome. We investigated if the dipstick measurement of albuminuria and creatinuria and the consequent ACR could be a reliable alternative to the more expensive and time-consuming reference methods used to diagnose microalbuminuria and follow up on kidney damage. Figure 1 reports a schematic diagram for sample preparation and testing.

### 2.1. Participant’s Selection and Study Design

From November 2020 to January 2021, we analyzed the clinical records of 249 individuals whose first-morning voids were analyzed at the Central Laboratory of Policlinico Umberto I University Hospital in Rome [19]. Subjects included: patients from different departments, including those with a high risk of CKD and patients with kidney transplantation; healthcare professionals on a routine check from occupational medicine; and external users of the laboratory. Participants were all indiscriminately included.

A summary of the characteristics of the participants is available in Table 1. This retrospective study was approved by the University Hospital ethical committee (Prot. 0620/2020), and all the study procedures were in accordance with the Helsinki Declaration of 1975, as revised in 1983, for human experimentation.

### 2.2. Data Collection

First-morning urine samples were collected in containers without conservatives using the hospital standard procedure and were analyzed the same morning of collection [20,21]. Every sample was analyzed first with the semi-quantitative method, then with the quantitative method.

### 2.3. Laboratory Examination

Quantitative analysis of albumin and creatinine was performed on a Cobas C 501 analyzer (Roche Diagnostics GmBH, Mannheim, Germany). Albumin in urine samples was measured using the bromocresol green colorimetric method. Urine creatinine levels were measured by an enzymatic colorimetric method that was ID/MS traceable. The ACR was calculated. A Sysmex UC-3500 automatic urine analyzer was used to evaluate ACR with a semi-quantitative method. For albumin, the dipstick analysis (Meditape UC-11A, Sysmex Corporation HQ: Kobe, Japan) is based on a PH indicator (tetra bromophenol blue); for creatinine, the method is based on the Benedict-Behre method.

### 2.4. Statistical Analysis

Statistical analysis was performed using GraphPad Prism v.5.01 (Boston, MA, USA), a commercial scientific 2D graphing and statistics software. We performed an initial descriptive analysis followed by a contingency analysis using the statistic tool. To analyze differences in categorical variables, especially those of a nominal nature, we used the chi-square (χ^2^) test and Fisher’s exact test [22]. To determine the statistical significance and measure of significance testing, we calculated the probability value concept (*p*-value) with a confidence interval (CI) of 95%. A *p*-value of 0.05 has been used as the cutoff for significance (statistically significant if *p* < 0.05).

## 3. Results

For results analysis, participants (n = 249) were divided into four different age groups: 59 patients were aged 0 to 19 (23.7%), 60 were aged 20 to 39 (24.1%), 62 were aged 40–64 (24.9%), and 68 were aged over 64 (27.3%). A total of 128 female patients (51.4%) and 121 male patients (48.6%) were included. This analysis, stratified for age and sex, represents a novelty with respect to previous studies [23].

### 3.1. Comparison between Albumin/Creatinine Ratio Calculated Using the Semi-Quantitative and the Quantitative Methods

We observed that the analysis performed on the Sysmex UC 3500 platform with reagent strips was not able to detect the ACR in 26 patients (10.4%). Indeed, for the urine diluted at the time of the dipstick analysis, the ACR ratio cannot be calculated. On the contrary, the quantitative test performed on the Cobas C 501 automatic analyzer successfully detected all the values. In the literature, diluted results are variously interpreted: they usually indicate that the urine is too diluted for an accurate calculation of the ACR because of an undetectable creatinine concentration on the strip test or a creatinine concentration of less than 50 mg/dL [24]. Interestingly, eleven samples that resulted diluted at the semi-quantitative method showed significant albuminuria (ACR above 30 mg/g) once re-analyzed with the quantitative assay. So, specimens that result in dilution with reagent strips require to be retested with quantitative methods to assess albuminuria, creatinuria, and ACR. Moreover, more than half (15) of the 26 patients with undetectable ACR belong to the groups aged under 20 or above 64 years old (Table 2).

The semiquantitative method was able to calculate ACR in 223 samples (89.6%) (Table 3). The quantitative test (Cobas C 501) revealed a negative ACR (<30 mg/g) for 141 subjects (63% of the 223 analyzed samples). Differently, for the semiquantitative method, only 107 patients had an ACR <30 mg/g (48%); 34 patients (15%) had discording values, with a larger number of subjects detected by the dipstick method with an ACR >300 mg/g. Quantitative testing showed 58 samples with an ACR between 30 and 300 mg/g (26%); differently, the semiquantitative method observed 68 samples (30%) in the same range, with some discordant samples.

The quantitative test reported only 24 subjects (11%) with a severe increase in urine albumin (ACR > 300 mg/g), while the dipstick assay observed a twice-high quote in the same range of 48 samples (22%), with an important overestimation. Of the overestimated 24 samples, according to the reference method, 2 have an ACR <30 mg/g and 22 have an ACR between 30 and 300 mg/g.

Specifically, there was a severe overestimation of the ACR, scoring a false positive for a severe increase in albumin excretion. Globally, the ACR was significantly overestimated by the semi-quantitative method compared to the quantitative one (*p*-value 0.001, χ^2^ 13.45).

Comparing the two methods in adults and pediatric subjects, it can be observed that the semi-quantitative method significantly overestimates ACR in patients aged 0–19 (*p*-value 0.002, χ^2^ 7.33), and 20–39 (*p*-value 0.008, χ^2^ 9.54), reflecting this way a more pessimistic clinical picture when compared to that of the quantitative method (Table 4). In these categories, the overestimation concerns almost exclusively the number of subjects with an ACR who comprehended between 30 and 300 mg/g. Even in patients aged >64 the semi-quantitative analysis overestimates albumin loss, with a larger quote of subjects over 300 mg with respect to the quantitative method (*p*-value 0.011, χ^2^ 8.96). However, no differences between the two methods were found in patients aged 40–64 years old.

By stratifying the population based on sex, we found that an overestimation of the ACR is present in both cases. The semi-quantitative method overestimates, importantly (by 50%), the number of female subjects with an ACR >300 mg/g. In male subjects, an overestimation occurs as well, but mainly for ACR levels comprised between 30 and 300 mg/g. In males and females, the percentage of subjects with normal ACR (<30 mg/g) is consequently underestimated. The differences between the two methods related to sex are both statistically significant if we compare the results of males and females as the sum of cases below 30 mg/g or over 30 mg/g (Fisher’s exact test *p*-values, respectively, 0.031 and 0.021) (Table 5).

### 3.2. Comparison between Albuminuria Analyzed Using the Semi-Quantitative and the Quantitative Methods

We categorized the results of albumin measured with MEDITAPE UC-11A strips as ≤30, ≤80 (but higher than 30), ≤150 (but higher than 80), or >150 mg/L. Considering these ranges of urine albumin concentration, we can see a discordance between the two methods at lower levels (below 80 mg/dL). The Cobas 501 assay reported 163 subjects belonging to the lowest urine albumin range: ≤30 mg/L; differently, the Sysmex UC 3500 analyzer scored 180 subjects in the same range, with an overestimation for the dipstick method.

**Figure 2:**
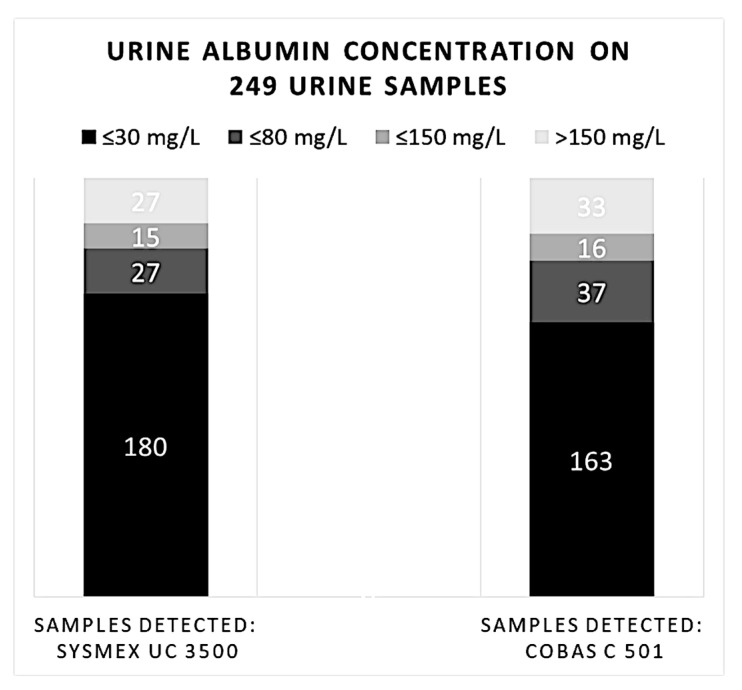
Comparison of 249 samples of albuminuria calculated using the semi-quantitative and quantitative methods and categorized on MEDITAPE UC-11A ranges (mg/L).

This overestimation goes at the expense of an underestimation by the Sysmex UC 3500 in the range ≤ 80 mg/L, where only 27 (69%) samples were located with respect to the 37 reported by the quantitative method. On the other hand, the semiquantitative method tended to be more consistent in the range > 150 mg/L, where it reached 96.4% concordance with the quantitative method. However, in the statistical analysis, no significant differences between the two methods were found (Figure 2).

**Figure 3:**
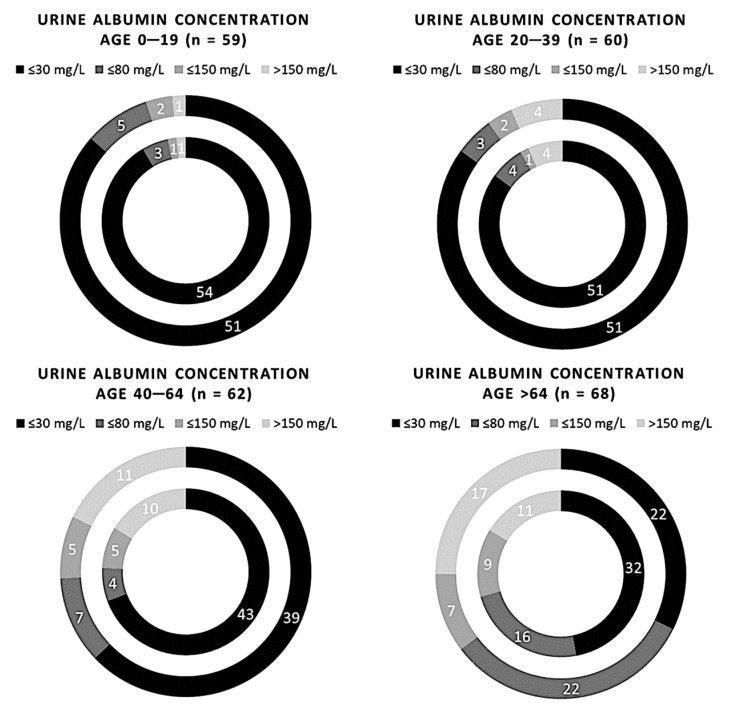
Comparison of 249 samples of albuminuria measured using the semi-quantitative and quantitative methods, redistributed on age. The inner circle represents the Cobas C 501 assay results, and the outer circle indicates the results of the Sysmex UC 3500.

**Figure 4:**
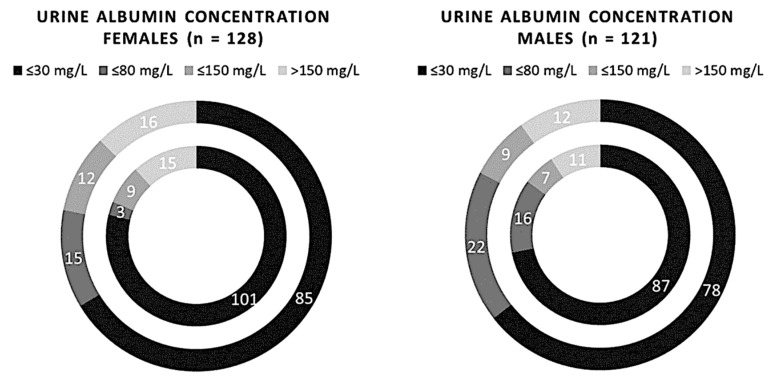
Comparison of 249 samples of albuminuria measured using the semi-quantitative and quantitative methods, redistributed on sex. The inner circle represents the Cobas C 501 assay results, and the outer circle indicates the results of the Sysmex UC 3500.

We can see how the semiquantitative method for albuminuria seems to progressively become less reliable going from younger people up to older people (Figure 3). On the other hand, no significant differences between the two methods were found in the statistical analysis (*p*-value > 0.05). Stratifying the risk based on sex, it was found that in female patients only, the semiquantitative method tends to underestimate albuminuria at the lowest range (*p*-value 0.02, χ^2^ 9.83) (Figure 4).

### 3.3. Comparison between Creatinuria Analyzed Using the Semi-Quantitative and the Quantitative Methods

**Figure 5:**
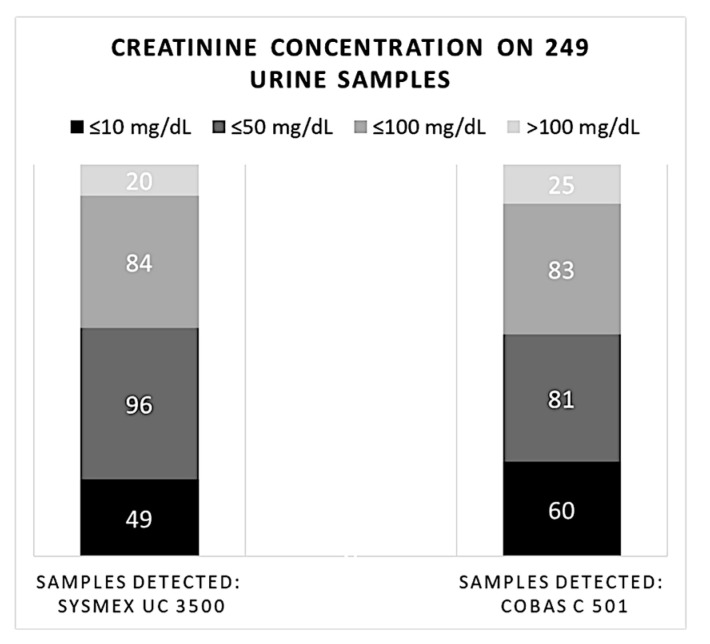
Comparison of 249 samples of creatinuria measured using the semi-quantitative and the quantitative methods categorized on MEDITAPE UC-11A ranges (mg/dL).

We categorized the results of creatinine measured with MEDITAPE UC-11A strips as ≤10, ≤50 (but higher than 10), ≤100 (but higher than 50), or >100 mg/dL. The semiquantitative method appeared quite reliable in the analysis of creatinuria. The reference method reported 60 samples with a value of urine creatinine ≤10 mg/dL (24.1%), 81 ≤ 50 mg/dL (32.5%), 83 ≤ 100 mg/dL (33.3%), and 25 samples with a value > 100 mg/dL (10.1%). Using the semi-quantitative method, we observed a concordance of 81.7% for the values ≤ 10 mg/dL (n = 49), a concordance of 84.4% for the ≤50 mg/dL range (n = 96), a discordance of 1.2 % (n = 84) for the ≤100 mg/dL range, and an 80% concordance for values > 100 mg/dL (n = 20). According to the reference method, values of urinary creatinine assessed by Sysmex UC 3500 comprised between 11 and 100 mg/dL and appeared slightly overestimated. No significant differences between the two methods were found in the statistical analysis (*p*-value > 0.05) (Figure 5).

**Figure 6:**
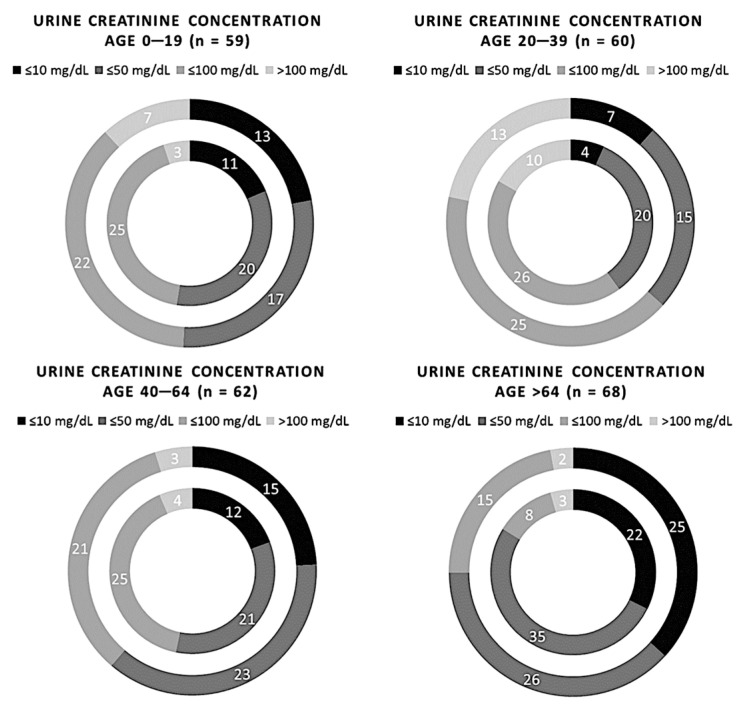
Comparison of 249 samples of creatinuria measured using the semi-quantitative and quantitative methods, redistributed on age. The inner circle represents the Cobas C 501 assay results, and the outer circle indicates the results of the Sysmex UC 3500.

**Figure 7:**
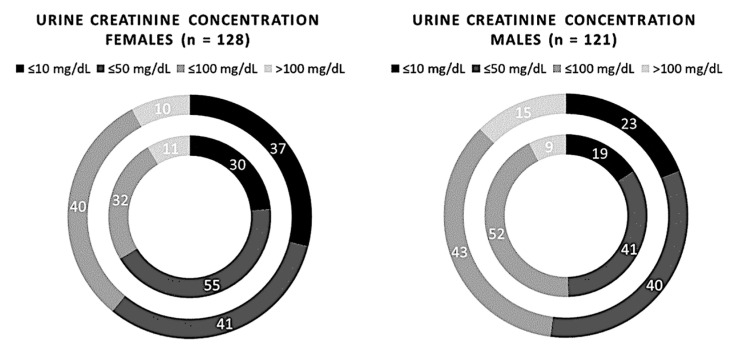
Comparison of 249 samples of creatinuria measured using the semi-quantitative and quantitative methods, redistributed on sex. The inner circle represents the Cobas C 501 assay results, and the outer circle indicates the results of the Sysmex UC 3500.

No significant differences between the two methods were found by stratifying the subjects based on age or sex (*p*-value > 0.05). Comparisons between the two methods by age are shown in Figure 6. The comparisons between the two methods in male and female subjects are shown in Figure 7.

## 4. Discussion

In this study, we show a comparison between a semiquantitative urinalysis method and quantitative assays for the assessment of urine creatinine, albumin, and ACR, with the aim to understand the reliability of the dipstick method as a preliminary screening diagnostic device to detect kidney disease at its stages. Moreover, for the first time, to the best of our knowledge, we present a deep and accurate analysis of the results, having performed a sub-stratification based on age (including pediatric subjects) and sex of the enrolled individuals for each parameter. [23]. In the literature, numerous large-scale studies have tried to set up new protocols for the laboratory diagnosis of albuminuria, creatinuria, and ACR, according to the necessity of a new, faster, and less expensive method useful for large-scale screening of the general population [25,26,27].

Evidence suggests that the screening of albuminuria, creatinuria, and ACR is especially important for patient stratification and for better management of CKD in its early stages; they are also powerful predictors of cardiovascular risk, reflecting a generalized atherosclerotic-mediated vasculopathy [28,29,30]. Furthermore, albuminuria, creatinuria, and ACR play a major role in the diagnosis, prognosis, and staging of renal damage in various diseases [31,32].

Our results show that the UC 3500 automatic urine analyzer is a valid method for ACR, albumin, and creatinine determination if used in the correct contexts. Our results showed a significant ACR overestimation of the semi-quantitative method compared to the quantitative method (*p*-value 0.001, χ^2^ 13.45); furthermore, this overestimation is more prominent in younger patients (aged under 39 years old). Moreover, by stratifying the risk based on sex, we found that the semi-quantitative method results in more reliable outcomes in male subjects when compared to female patients. The age and sex discrepancies observed between the semi-quantitative and quantitative tests could be due to difficult and/or incorrect urine sample collection.

In fact, the most suitable type of specimen is represented by midstream samples of first-morning urine, free of contaminants (including soap), collected in a sterile container (preferably vacuum-packed) after cleaning the genital area, and delivered to the laboratory as soon as possible (within 2 h). A preliminary step, represented by the correct hygiene of the hands, urethra, and external genital tract using soap and water followed by drying before sample collection, is mandatory. Moreover, a recent intake of antibiotics may affect the test outcome. In women of childbearing age, the sample should be collected 3–4 days after the end of menstruation. Correct sample collection in women can be difficult, and contamination from genital secretions due to the anatomic relationship between the two systems is not rare.

Even in young pediatric patients, there may be difficulties in the sample collection, especially for those children who do not control urination. In this case, it is possible, after thorough washing, to place a sterile adhesive bag on the genital area; however, this method brings higher levels of contamination. The quantitative test can overcome some of these problems by undergoing specimen centrifugation before testing, especially when cloudy samples are delivered to the laboratory.

Contaminated and diluted samples are very common, so information and a training campaign seem important, especially for younger people. Higher ACR values from the semiquantitative method, especially in the case of younger populations and female patients, should be subsequently monitored with the quantitative method. Sex differences should be further evaluated by the industry when benchmarks are established [33]. As shown in the results, the ACR cannot be performed in urine due to the dilution caused by the semi-quantitative method, and samples should be re-analyzed with quantitative assays to obtain the ACR values (Table 2). With the aim of a general, fast population screening, this could be the limit of this test. In the case of diluted urine samples, a quantitative test should be mandatory.

The dipstick method at best gave a reliability of 96% (compared to the quantitative method) for albuminuria. The best reliability was found when albuminuria was >150 mg/L, but a great concordance between the two methods was found also at the lowest levels (90.5% at values below 30 mg/L), suggesting that the dipstick is a feasible diagnostic device for monitoring and management. Indeed, therapeutic strategies aimed at reducing albuminuria and ACR are associated with protection and lower mortality for diabetic patients, preventing the severe development of cardiovascular events and kidney damage [34]. Numerous large cohort studies have analyzed the correlation between albuminuria and ACR and the mortality rate in the general population [35,36,37,38]. Some have even suggested that the semiquantitative method has high sensitivity and negative predictive value (NPV), which are beneficial for laboratory screening in both albuminuria and proteinuria [24]. It is important in the future to develop a prevention strategy for microalbuminuria, in order to prevent the onset of kidney disease [39].

The range of ACR between 30 and 300 mg/g is important to diagnose “microalbuminuria”, a moderate increment of albumin urinary excretion that can predict kidney and cardiovascular outcomes in patients. Assuming to propose the semiquantitative method as a large population screening strategy to diagnose early kidney damage, we found a good correlation with the quantitative laboratory method. For patients with microalbuminuria (ACR 30–300 mg/g) or severe albuminuria excretion (ACR > 300 mg/g), a valid diagnostic strategy should be to test them with the quantitative method, to confirm the value, to have a more precise calculation of the ACR, and to discriminate microalbuminuria’s range from severe albumin loss.

## 5. Conclusions

As observed, the urinalysis test trend is to overestimate the ACR’s value, resulting in a higher number of false positives compared to the reference method. These results are in line with a good screening method, but positives need to be confirmed with quantitative methods, especially (but not exclusively) in females and younger people. Diluted samples need to be re-analyzed by quantitative methods too. The Sysmex UC 3500 proposes a semi-quantitative method to calculate the ACR along with the creatinuria and albuminuria values in first-morning urine samples with promising results for extensive screening program application: less expensive, faster, and less difficult for the patient to withdraw. According to our study, the semiquantitative dipstick test is a valid laboratory method, mainly for an apparently healthy population, to predict early renal damage and prevent cardiovascular risk. This method can act as a first-level analysis, which should flank but not replace the gold standard test (the quantitative method), which should be used to confirm the ACR’s value in the case of pathological results.

## Figures and Tables

**Table 1:** Characteristics of the 249 included individuals involved in the study. Departments, number of included subjects per department (n), age (mean, range), male/female patient ratio (M/F).

Hospital Departments	n (249)	AGE	M/F
Occupational Medicine	22	35 (28–60)	7/15
Gastroenterology	5	58 (30–74)	4/1
Daily Surgery	1	46	0/1
Transplants	31	54 (4–74)	22/9
Cardiology	5	71 (56–90)	3/2
Intensive Care Unit	5	50 (35–60)	4/1
Neurosurgery	11	57 (25–87)	8/3
Pediatrics	58	13 (1–24)	27/31
Radiotherapy	2	80 (72–88)	2/0
Infectious Diseases	19	67 (45–87)	11/8
Internal Medicine	30	69 (14–96)	12/18
Pneumology	8	58 (30–89)	6/2
Clinical Immunology	3	64 (38–84)	3/0
Diabetology and Obesity	5	17 (7–29)	3/2
Tropical Diseases	3	67 (49–89)	1/2
Rheumatology	11	66 (29–92)	2/9
Geriatrics	2	76 (70–81)	2/0
Physical Medicine and Rehabilitation	2	83 (82–84)	0/2
Nephrology	1	35	0/1
Gynecology and Obstetrics	18	33 (23–53)	0/18
Neurology	2	87 (82–92)	1/1
Laboratory External Users	5	52 (34–62)	3/2

**Table 2:** In 26 patients, the semi-quantitative method (Sysmex UC 3500) with a urinary dipstick was not able to detect the ACR, while the quantitative test (Cobas C 501) successfully detected all the values.

Patient n.	Sex M/F	Age	Ratio Sysmex	Ratio Cobas (mg/g)
15	6/9	34 (1–81)	ND	<30
9	5/4	47 (11–90)	ND	30–300
2	F	85 (74–96)	ND	>300

**Table 3:** Comparison of 223 samples of ACR calculated using the semi-quantitative and quantitative methods. Sysmex UC 3500 was not able to detect ACR in 26 of the 249 analyzed samples.

Ratio Range (mg/g)	Sysmex UC 3500 (n)	Cobas C 501 (n)
<30	107	141
30–300	68	58
>300	48	24
Tot.	223	223

**Table 4:** Comparison of 223 samples of albumin/creatinine ratio calculated using the semi-quantitative and quantitative methods, redistributed by age.

Age	Sysmex UC 3500 (mg/g→n)	Cobas C 501 (mg/g→n)
0–19 (n = 50)	<30→33 30–300→16 >300→1	<30→44 30–300→5 >300→1
20–39 (n = 56)	<30→35 30–300→17 >300→4	<30→48 30–300→6 >300→2
40–64 (n = 56)	<30→26 30–300→13 >300→17	<30→32 30–300→12 >300→12
>64 (n = 61)	<30→13 30–300→22 >300→26	<30→17 30–300→35 >300→9

**Table 5:** Stratification of urinalysis results of albumin/creatinine ratio based on sex.

Sex	Sysmex UC 3500 (mg/g→n)	Cobas C 501 (mg/g→n)
Female (n = 113)	<30→56 30–300→27 >300→30	<30→73 30–300→27 >300→13
Male (n = 110)	<30→51 30–300→41 >300→18	<30→68 30–300→30 >300→12

## Data Availability

Data available on request.
